# Drugs during Pregnancy and Lactation: New Solutions to Serious Challenges

**DOI:** 10.1155/2012/206179

**Published:** 2012-03-19

**Authors:** Gideon Koren, Shannon Clark, Doreen Matsui

**Affiliations:** ^1^Department of Pediatrics, University of Western Ontario, London, ON, Canada N6A 5W9; ^2^Division of Clinical Pharmacology and Toxicology, Hospital for Sick Children, 555 University Avenue, Toronto, ON, Canada M5G 1X8; ^3^University of Texas, Galveston, TX 77555, USA

Ethically, it is very difficult to use the classical paradigms of drug studies in pregnancy, due to the potential fetal risks of prospective exposure of the mother and fetus to chemicals for which safety has not been confirmed. This special issue presents new approaches and offers novel solutions to serious challenges, from the use of antidepressants in pregnancy to domperidone in enhancing lactation, from the potential fetal advantages of polyunsaturated fatty acids to the potential risk of drugs of abuse.

The majority of pregnant women use medications during pregnancy either before recognizing they have conceived or due to the need to treat medical conditions that may affect maternal and fetal well-being. Due to the potential fetal risks of drugs, very few randomized controlled trials are being conducted in early pregnancy, and the numbers are scarce even after embryogenesis has been completed. As a result, women are typically orphaned from the benefits of new therapeutic modalities. Yet, with women tending to postpone the age of starting a family, substantially larger numbers of women experience chronic conditions that necessitate drug therapy.

This issue focuses on major challenges in the area of drug therapy during pregnancy and lactation, looking for evidence of safety and effectiveness with the use of a variety of research methodologies that try to replace the gold standard of randomized clinical trials.

O. Diav-Citrin and A. Ornoy tackle what is probably the biggest contemporary controversy—the fetal safety of antidepressants. Through painstaking review of over 30 studies, they come to the conclusion that fetal and neonatal risks, even if they exist, occur at very low rates and are outweighed by the maternal benefits of these drugs.

M. Moretti and colleagues focus on another controversial area—the fetal safety of ACE inhibitors, commonly used for hypertension. By including a control group matched for the hypertensive morbidity, they strengthen the growing body of evidence suggesting no increased fetal risks after first trimester exposure to ACE inhibitors. With convincing evidence that neonatal folic acid supplementation and fortification can prevent neural tube defects, a fear has emerged that too much circulating folate may increase the risk of cancer as an adverse effect of unmetabolized folic acid. C. Tam and colleagues document that levels of unmetabolized folic acid do not increase in women even when taking 5 mg daily. This detailed biochemical study adds important reassurance that short period use of even 5 mg folate (which is needed in groups of women at high risk for neural tube defects) is safe to the expecting mother.

S. M. Clark and colleagues update the reader on the advances in treating the most common condition in pregnancy—morning sickness. Even today, a large proportion of pregnant women are afraid to treat the symptoms of nausea and vomiting of pregnancy due to unjustified perception of fetal risks. S. M. Clark et al. match the evidence of effectiveness with that of fetal safety, documenting that in 2012 there is no logical reason for a woman not to be managed safely for this condition.

Breastfeeding is the ideal method of infant nutrition; however, not rarely women cannot establish effective milk flow to ensure optimal feeding. Two research papers in this special issue address this problem, relating directly to the dopaminergic agent domperidone. A. Osadschy and colleagues use a meta-analytic technique to provide evidence that the existing, small studies prove the effectiveness of the drug in improving milk production. C. Mannion et al., bringing the important angle of lactation consultants, explore in a pilot study determinants of success and failure in establishing breastfeeding.

Three papers in this issue explore in-depth methodological issues which challenge different aspects of drug therapy in pregnancy. B. Källén brings his many years of experience in pharmacoepidemiology to the critical discussion of confounders which may affect the interpretation of administrative data bases. These prescription databases, when linked with databases of pregnancy outcome, create a potentially powerful tool in exploring fetal safety of drugs. However, this method is challenged by a large number of serious confounders that must be considered and addressed.

D. Matsui tackles one of the most serious issues in drug therapy in pregnancy—patient compliance. Due to fears of teratogenicity, expecting women tend not to take their medications as prescribed, even in cases of life-threatening conditions. Deeper understanding of these issues is critical if we are to improve drug therapy in pregnancy.

One of the biggest ethical issues in pregnancy is how to gain knowledge on fetal exposure to drugs, when many of these drugs may be unsafe. C. Gedeon and colleagues explore the ethical and practical implications of giving the mother a drug just before an elective pregnancy termination and measuring its kinetics in the abortus.

It appears that scores of such studies have been conducted by scientists in different countries, but this practice has not been accompanied by in-depth discussion on its ethical and legal implication.

Over the last 2 decades a large body of experimental animal research has documented the role of polyunsaturated fatty acids (PUFAs) in ensuring normal fetal brain development. This research has been mirrored by human studies suggesting that offspring of women who suffer from deficits in PUFA are lagging in their visual development. This has led to the commonly held suggestion that enriching the diets of healthy women who do not exhibit PUFA deficiency may improve offspring brain function. Lo and colleague offer the first systematic review of all randomized studies in pregnancy looking at the potential benefits of PUFA supplementation. The results bring into question voices calling for sweeping recommendation to supplement with PUFA women who enjoy normal nutrition.

Lastly, a unique paper by Unger and colleagues tackles the issues of drug exposure and therapy for women suffering from addiction. With increasing numbers of drug-dependent women of reproductive age, optimal care of these women and their unborn children, neonates, and infants should be a major focus.

We hope that you, the reader, will find this special issue stimulating and, as important, relevant to your practice.

*Gideon Koren* *Gideon Koren* *Shannon Clark* *Shannon Clark* *Doreen Matsui* *Doreen Matsui*

